# PLA Nanofibers for Microenvironmental-Responsive Quercetin Release in Local Periodontal Treatment

**DOI:** 10.3390/molecules27072205

**Published:** 2022-03-28

**Authors:** Francesca Di Cristo, Anna Valentino, Ilenia De Luca, Gianfranco Peluso, Irene Bonadies, Anna Calarco, Anna Di Salle

**Affiliations:** 1 Elleva Pharma s.r.l., Via Pietro Castellino 111, 80131 Naples, Italy; francesca.dicristo@ellevapharma.com; 2 Research Institute on Terrestrial Ecosystems (IRET)—CNR, Via Pietro Castellino 111, 80131 Naples, Italy; anna.valentino@iret.cnr.it (A.V.); ilenia.deluca@iret.cnr.it (I.D.L.); gianfranco.peluso@unicamillus.org (G.P.); anna.disalle@cnr.it (A.D.S.); 3 Faculty of Medicine and Surgery, Saint Camillus International University of Health Sciences, Via di Sant’Alessandro 8, 00131 Rome, Italy; 4 Institute for Polymers, Composites and Biomaterials (IPCB-CNR), Via Campi Flegrei 34, 80078 Pozzuoli, Italy

**Keywords:** quercetin, pH-responsive, electrospinning, periodontitis, inflammation

## Abstract

The management of periodontitis remains a vital clinical challenge due to the interplay between the microorganisms of the dental biofilm and the host inflammatory response leading to a degenerative process in the surrounding tissues. Quercetin (QUE), a natural flavonol found in many foods, including apples, onions and tea, has exhibited prolonged and strong antibiofilm and anti-inflammatory effects both in vitro and in vivo. However, its clinical application is limited by its poor stability and water solubility, as well as its low bioavailability. Thus, in the present study, electrospun polylactic acid (PLA) nanofibers loaded with different amounts (5–10% *w*/*w*) of QUE were produced to rapidly respond to the acidic microenvironment typical of periodontal pockets during periodontal disease. This strategy demonstrated that PLA-QUE membranes can act as a drug reservoir releasing high QUE concentrations in the presence of oral bacterial infection (pH < 5.5), and thus limiting *Pseudomonas aeruginosa* PAO1 and *Streptococcus mutans* biofilm maturation. In addition, released QUE exerts antioxidant and anti-inflammatory effects on *P. gingivalis* Lipopolysaccharide (LPS)-stimulated human gingival fibroblast (HGFs). The reported results confirmed that PLA-QUE membranes could inhibit subgingival biofilm maturation while reducing interleukin release, thereby limiting host inflammatory response. Overall, this study provided an effective pH-sensitive drug delivery system as a promising strategy for treating periodontitis.

## 1. Introduction

Periodontitis is a chronic multifactorial inflammatory disease characterized by the progressive destruction of gingival connective tissue and alveolar bone which, if left untreated, leads to irreversible tooth loss [1,2]. Much evidence suggests that the onset and propagation of this periodontal disease occurs through a dysbiosis of the commensal oral microbiota forming the biofilm, as well as the host’s immune responses [3,4]. In addition, reactive oxygen species (ROS) produced by immune cells cause the aggravation of gingival tissue injury. Numerous international studies report the association of periodontitis with other chronic inflammation-driven disorders, including cardio-metabolic, autoimmune diseases, and cancer [5,6].

Current non-surgical strategies to treat periodontal disease focus on the elimination of the active disease by mechanical plaque removal or local antimicrobial therapy. However, these approaches result in modest and mostly temporary success. In particular, local treatment with antibiotics is insufficient since they lack effective drug concentrations at the target sites. On the other hand, systemic treatment with antibiotics from prolonged use can induce the development of bacterial resistance [7,8,9]. 

In this framework, the attention of the scientific community is shifting toward adjuvants to conventional periodontal treatments that have no antibiotic resistance and few side-effects, such as polyphenols. Polyphenols are promising bioactive micronutrients with antibacterial, anti-inflammatory and antioxidant effects, acting as potential drugs in modern biomedicine [10,11]. 

Among them, quercetin (QUE), a flavonoid compound ubiquitously present in roots and leaves of various vegetables, has a plethora of beneficial effects on human health, including in preventing and treating various systemic and oral diseases [12,13]. 

Moreover, previous reports have found that quercetin can inhibit the release of inflammatory cytokines in human gingival fibroblasts (HGFs) stimulated by *Porphyromonas gingivalis* lipopolysaccharide in vitro and alleviate alveolar bone resorption in in vivo animal models [14,15,16,17]. Although several studies identify QUE as a promising phytochemical to sustain periodontal tissue health, the poor bioavailability, high metabolic rate, and rapid clearance from body of QUE limit its clinical application [18]. Therefore, the development of local delivery platforms that would overcome the biopharmaceutical obstacles and avoid systemic clearance of QUE, thereby increasing its therapeutic and prophylactic potential, is still a technological challenge. In this context, electrospinning has emerged as a promising technique for the fabrication of delivery carriers to treat different types of oral diseases, including periodontitis, providing a good microenvironment for limiting bacterial and biofilm adhesion and promoting bone and tissue regeneration [19,20,21,22]. Moreover, since periodontal disease is mainly confined to the periodontal pocket, electrospun membranes can adhere to the gingival tissue, loading the drug in tight contact with surrounding tissue and prolonging the therapeutic effect. Although QUE was shown to exhibit positive effects for the treatment of periodontal disease, to the best of our knowledge, an appropriate delivery system assessing the effect of QUE on inflammation and the oral biofilm simultaneously during periodontitis has not yet been described.

In this paper, poly(DL-lactic acid) (PLA) nanofibers loaded with different amounts of QUE were produced and characterized and their antibiofilm and inflammatory potential evaluated. The results reported herein demonstrate a release kinetic strongly affected by the pH of the medium. This reactivity to pH variations (from neutral to slightly acidic), such as those occurring in the oral cavity under pathological conditions [23], allowed us to obtain a local delivery platform capable of counteracting the maturation of the biofilm when the pH of the oral environment is lower than 5.5. In addition, the released QUE can have strong antioxidant and anti-inflammatory effects on an in vitro-induced inflammatory environment mimicking periodontal disease.

Taken together, the reported data suggest that PLA-QUE fibers could be administrated into the periodontal pocket as promising local adjuvants to simultaneously control inflammation and oral microbiome maturation in periodontal disease. The delivery of the bioactive molecule directly to the periodontal pocket decreases the dose and application frequency and increases patient compliance, overcoming the current therapeutic shortcomings.

## 2. Experimental Section

### 2.1. Materials

PLA (Ingeo 4032D) with 0.7 mol% L-isomer, with a weight average molar mass of 210 kDa and a polydispersity (PDI) = 1.7, was supplied by NatureWorks LLC. Chloroform (CHL), N,N-Dimethylformamide (DMF), ethanol and acetone with a purity ≥ 99.8% were purchased from Sigma-Aldrich and used without further purification. 

Quercetin, DPPH (2,2-diphenyl-1-picrylhydrazyl), 2′,7′-dichlorofluorescein diacetate (DCFH-DA), Lipopolysaccharide from *Porphyromonas gingivalis* (LPS), a Lipid Peroxidation (MDA) Assay Kit, a Ferric Reducing Antioxidant Power (FRAP) Assay Kit and 3-(4,5-Dimethylthiazol-2-yl)-2,5-diphenyl tetrazolium bromide (MTT) were purchased from Sigma-Aldrich Co. (Milan, Italy). *Streptococcus mutans* (ATCC^®^ 25175), *Pseudomonas aeruginosa* PAO1 (ATCC^®^ BAA-47™), immortalized human gingival fibroblast (HGF, ATCC CRL-2014) were purchased from the American Type Culture Collection (ATCC, Milan, Italy) and cultured following the ATCC’s guidelines. The cells were maintained in Dulbecco Modified Eagle Medium (DMEM) high-glucose supplemented with 10% Fetal Bovine Serum (FBS), and 1% penicillin/streptomycin at 37 °C in a 5% CO_2_ atmosphere. When the cell confluency reached 80–90%, a subculture was performed at 2 × 10^5^ cells/mL using trypsin-EDTA (0.05%; Euroclone). The cells were regularly tested for *Mycoplasma* contamination.

### 2.2. Preparation of Electrospinning Solutions and Membrane Manufacturing

Electrospun membranes were realized starting from a PLA solution containing different amounts of quercetin. Neat PLA solutions (coded as PLA) were prepared by dissolving 10% (*w*/*w*). PLA in chloroform/dimethylformamide (CHL/DMF, 80/20 *v*/*v*); after that, QUE (5% *w*/*w* and 10% *w*/*w* respect to the PLA) was directly added to the polymer solutions and stirred with a magnetic mixer for at least 6 h. The obtained electrospun membranes were coded as PLA-QUE_5_ and PLA-QUE_10_. 

The PLA solutions were electrospun with NANON01 equipment (MECC Co., Ltd. Fukuoka, Japan), by using a single nozzle and a plate collector, at 25°C and 10% relative humidity. After optimization of the process parameters, the flow rate was fixed at 2 mL h^−1^, and the applied voltage and the distance between the nozzle and the collector adjusted to 25 kV and 30 cm, respectively. 

### 2.3. Characterization of Electrospun Membranes

The morphology of the membranes was evaluated using a FEI Phenom Desktop scanning electron microscope (Eindhoven, The Netherlands). Before analysis, the samples were sputtered/coated with an Au–Pd alloy using a Baltech Med 020 Sputter Coater System and then mounted on aluminum stubs. The average fiber diameter distribution was analyzed using the ImageJ software (NIH, Bethesda, MD, USA).

The chemical composition of the membranes was investigated by means of Fourier transform infrared spectroscopy coupled with the attenuated total reflectance technique (ATR-FTIR). The spectra were acquired in the spectral region between 4000 and 400 cm^−1^. The analysis was performed using the Origin software (OriginPro 8 SR0, OriginLab Corporation, Northampton, MA, USA). The QUE spectrum was considered the positive control. 

### 2.4. Encapsulation Efficiency and In Vitro Drug Release Assay

The encapsulation efficiency (EE) of QUE in the nanofibers was quantified by dissolving the QUE-encapsulated nanofibers in methanol, and the amount of released QUE was measured by HPLC-UV with an isocratic elution consisting of mobile phase A (0.3% trifluoracetic acid) and B (acetonitrile/water 50:50 *v*/*v*). The detection wavelength was set at 254 nm and QUE quantification was based on a standard curve in phosphate-buffered saline (PBS). Before injection, the standards and samples were filtered through a 0.22 µm pore-size filter (Millipore, Milan, Italy). System control and data acquisition were performed using the ChemStation software 4.03 on 27 January 2020 (Agilent Technologies, Milan, Italy). The amount of QUE in the fibers was calculated from the obtained data against a predetermined calibration curve of the drug (1 mg/mL). The encapsulation efficiency of the QUE was determined as follows in Equation (1):

(1) $$\mathrm{EE}\;\left(\%\right)=\frac{\mathrm{quercetin}\;\mathrm{amount}\;\mathrm{found}\;\mathrm{in}\;\mathrm{nanofibers}\;\left(\mathrm{mg}\right)}{\mathrm{quercetin}\;\mathrm{amount}\;\mathrm{initially}\;\mathrm{added}\;\mathrm{in}\;\mathrm{the}\;\mathrm{polymer}\;\mathrm{solution}\;\left(\mathrm{mg}\right)}\;$$

The QUE release was investigated as reported by Bonadies et al., with some modifications [24]. Briefly, electrospun membranes of similar weight were placed into individual vials covered with aluminum foil to preserve the drug from degradation caused by light. The release kinetics were performed at 37 °C in artificial saliva medium (SAGF) containing 4 g of sucrose (SAGF-suc). The pH was adjusted to 4.8 and 6.8 with HCl and NaOH, respectively. At predetermined time intervals (every hour for the first 8 h, then every 24 h over 120 h), supernatants were withdrawn, and the same amount of fresh solution was added back to the release medium to maintain the sink condition. The QUE concentration was measured as reported above by HPLC-UV. The results were presented in terms of cumulative release as a function of time.

### 2.5. Antioxidant Studies

The antioxidant activity of the PLA-QUE membranes was determined using DPPH assay, as reported by Stoyanova et al. [25]. Similar pieces of membranes (ca. 30 mm diameter) were immersed in a 2 mL of DPPH ethanolic solution (60 µM) and incubated under dark conditions at 20 °C for 30 min. The absorbance of the samples was determined at 517 nm using a microplate reader (Cytation 3, AHSI, Milan, Italy). The percentage of inhibition of DPPH was determined by the formula in Equation (2):

(2) $$\mathrm{DPPH}\;\mathrm{scavenging}\;\mathrm{effect}\;\left(\%\right)=\frac{A_{1}-A_{0}}{A_{1}}\times \;100$$

where A_1_ was the absorbance of the pure ethanolic DPPH and A_0_ that of the membrane-incubated DPPH solution. The antioxidant activity was expressed as % with respect to free quercetin. All experiments were performed in triplicate.

A ferric reducing antioxidant power (FRAP) assay was performed to determine the capacity of synthesized membranes to reduce Fe^3+^ to Fe^2+^. The FRAP method was conducted as reported by Amrati et al., with slight modifications [26]. As described above, the membranes were dissolved in artificial saliva for 3, 6, 12 and 24 h, ultrasonicated for 15 min and then incubated at 50 °C for 20 min in the presence of potassium ferricyanide (1%). Afterward, trichloroacetic acid (10%) was added and the obtained solutions were centrifuged for 10 min at 930 rcf. Finally, the supernatant was combined with FeCl_3_ (0.1%) and distilled water and the absorbance was spectrophotometrically measured at 700 nm. 

### 2.6. Antibacterial Studies

#### 2.6.1. Antimicrobial Activity

The antimicrobial efficacy of QUE-loaded membranes was assessed by monitoring the inhibition of *Pseudomonas aeruginosa* PAO1 and the growth rate of *Streptococcus mutans (S. mutans)*, as previously described with some modifications [27]. Briefly, an electrospun membrane of similar dimensions was sterilized by UV radiation for 15 min at each side, placed in a 12-well plate, covered with 500 μL of liquid broth supplemented with 20% sucrose and inoculated with a bacterial suspension containing a microbial concentration of approximately 1 × 10^7^ CFU/mL. The plate was then incubated at 37 °C and 200 rpm in a microplate reader (Cytation 3) and, at pre-established times (9, 24, 48 and 96 h), the bacterial suspensions were subjected to turbidity analysis by recording the optical density (OD) at 600 nm. In addition, the pH of the bacterial cultures was also monitored using a pH electrode (Mettler-Toledo, Milan, Italy).

The medium without the bacterial solution and the bacterial solution without the membranes were set as blank and negative controls, respectively. The experiments were performed as triplicates.

#### 2.6.2. Biofilm Analysis

The ability of the QUE-loaded membranes to inhibit biofilm formation was analyzed under both static and dynamic conditions.

The crystal violet (CV) biofilm assay was used to determine bacterial biofilm formation under static conditions, as previously described [19]. Briefly, pieces of membranes of similar size were sterilized by UV radiation for 15 min at each side, placed in a 48-well polystyrene plate, inoculated with 750 μL of liquid medium broth containing PAO1 (1 × 10^7^ CFU/mL) or *S. mutans* (1 × 10^7^ CFU/mL) and cultured without shaking at 37 °C in a humid atmosphere. PLA mat incubated in liquid medium broth was used as a negative control, while PAO1 (1 × 10^7^ CFU/mL) and *S. mutans* (1 × 10^7^ CFU/mL) were used as positive controls. After 9, 24 and 48 h, the surface-adhered biofilm was gently washed with sterile PBS, stained with 0.1% *w*/*v* CV and dissolved in 96% ethanol. Biofilm formation was then quantified by measuring the absorbance at 570 nm (OD570) in a microplate reader (Cytation 3). Measurements were carried out in triplicate for each membrane.

In addition, biofilm viability was evaluated under dynamic conditions and in the presence or absence of salivary pellicle by an MTT assay using the drip-flow reactor (M-DFR) previously described [28]. Briefly, salivary pellicle formation was obtained by covering the disks’ surfaces with thawed sterile saliva and incubating at 37 °C for 24 h. Successively, the supernatant was carefully removed. Biofilm was developed by growing each bacterial suspension in the DFR flow cells for 4 h to allow for bacterial adhesion. Then, a constant flow (9.0 mL/h) of Nutrient Broth (NB, Oxoid, Basingstoke, Hants, UK) was provided using a peristaltic pump and the temperature maintained at 37 °C. After 48 or 96 h of incubation, the disks were removed, immediately washed with sterile PBS to remove nonadherent bacteria and placed in a 12-well plate. To determine biofilm viability, 500 μL of 0.3 mg/mL of MTT was added to each well and incubated at 37 °C in the dark. After 3 h, the MTT solution was removed, and the formed formazan crystals were dissolved by adding 500 μL dimethyl sulfoxide (DMSO). OD510 nm was recorded using a microplate reader.

#### 2.6.3. Quorum Sensing (QS) Interfering

The capability of PLA-QUE membranes to interfere with the quorum sensing mechanism that guides the biofilm maturation process was evaluated by real-time PCR (qRT-PCR) quantifying the mRNA level of rhlAB genes for PAO1 and of comCD genes for *S. mutans*, as previously described [19]. PAO1 and S. mutans biofilm were developed in the presence of synthesized membranes for 12 h in a 48-well polystyrene plate. Then, total RNA was extracted using TRIzol reagent (Invitrogen, Milan, Italy) and underwent retro transcription using AMV reverse transcriptase and random hexamers according to the provider’s instruction (Promega, Milan, Italy). The resulting mixture was amplified by qRT-PCR using specific primers based on our previous work, as listed in Table 1. 

### 2.7. In Vitro Cell Studies

#### 2.7.1. Oxidative Stress and Lipid Peroxidation

The production of ROS by HGFs after infection with *P. gingivalis* -LPS was measured by a DCFH-DA assay according to the manufacturer’s protocol. HGF cells (3 × 10^5^ cells/well) were seeded in 24-well black tissue culture plates with transparent bottoms and were left to settle for 24 h at 37 °C under 5% CO_2_. Then, the cells were preincubated in the presence of QUE-loaded membranes for 24 h before the addition of 1 μg/mL LPS (Invivogen) to mimic the oxidative stress status in periodontitis. After the incubation period, the membranes were removed and the cells incubated with DCFH-DA (25 μM) for 1 h in the dark. The fluorescence was measured every 5 min for 1 h using a microplate reader (Cytation 3) with an excitation wavelength of 485 nm and an emission wavelength of 535 nm. 

To assess the lipid peroxidation, the malondialdehyde (MDA) concentration was determined using the thiobarbituric acid reactive substances (TBARS) assay, according to the manufacturer’s protocol. This test is based on the reaction of MDA, a breakdown product of lipid peroxides, with thiobarbituric acid. The basal concentration of MDA was established by adding 600 µL of TBARS solution to 50 µg of total protein dissolved in 300 µL of Milli-Q water. The mix was incubated for 40 min at 100 °C prior to centrifugation at 14,000 rpm for 2 min. The supernatant was analyzed spectrophotometrically at 532 nm with a microplate reader. 

#### 2.7.2. Anti-Inflammatory Activity

The inflammatory activity of PLA-QUE membranes was evaluated by real-time quantitative PCR (RT-qPCR). HGFs were seeded in a 6-well plate at a density of 1 × 10^6^ cells/well and pretreated with membranes for 24 h, followed by LPS stimulation for 3 h. Then, total RNA was extracted from the HGFs using TriFast (EuroClone, Milan, Italy), and according to the manufacturer’s protocol, cDNA was synthesized using the Wonder RT cDNA Synthesis Kit (EuroClone). Next, mRNA levels of IL-1α, IL-1β, IL-6, IL-8 and TNF-α were evaluated by 7900HT Fast Real-Time PCR System (Applied Biosystem, Milan, Italy). The reactions were performed according to the manufacturer’s instructions using SYBR Green PCR Master Mix (Euroclone). The mRNA levels of these targets were calculated using the 2^−ΔΔCt^ method and normalized against β-actin, which was used as an internal reference gene. The results were expressed as fold changes to control. The primer information for related mRNAs is shown in Table 2.

### 2.8. Statistical Analysis

The results were expressed as mean ± standard deviation (SD). Student’s *t*-test was used for quercetin release. One-way analysis of variance (ANOVA) and Tukey’s post-hoc test for statistical comparison were used for an antimicrobial investigations assay, as well as quantitative real-time PCR. The difference was considered statistically significant when *p* < 0.05. All the data were analyzed using the GraphPad Prism version 6.01 statistical software package (GraphPad, San Diego, CA, USA).

## 3. Results and Discussion

### 3.1. Membrane Characterization

Electrospun nanofibers loaded with natural bioactive molecules such as QUE are largely investigated as biomedical devices due to the ability of nanofibers to enhance the bioavailability and the therapeutic efficiency of drug playload by providing a means of sustained release [29]. Among the polymers used as a matrix for the electrospinning process, synthetic poly (lactic acid), poly(lactide-co-glycolide) (PLGA) and polycaprolactone (PCL) were the most widely used. For example, Ajmal et al. developed nanofibers of PCL containing ciprofloxacin hydrochloride and QUE able to protect the red blood cell membrane against lipid peroxidation, helping to maintain its functionality [30]. In another study, Vashisth et al. [31] produced an implantable anticancer drug using QUE loaded onto PLGA/PCL to increase its bioavailability. The release profiles of QUE from the nanofibers in phosphate-buffered saline showed controlled release of the biomolecule up to 120 h, demonstrating an anticancer effect against human hepatocellular carcinoma (HepG2). 

**Figure 1:**
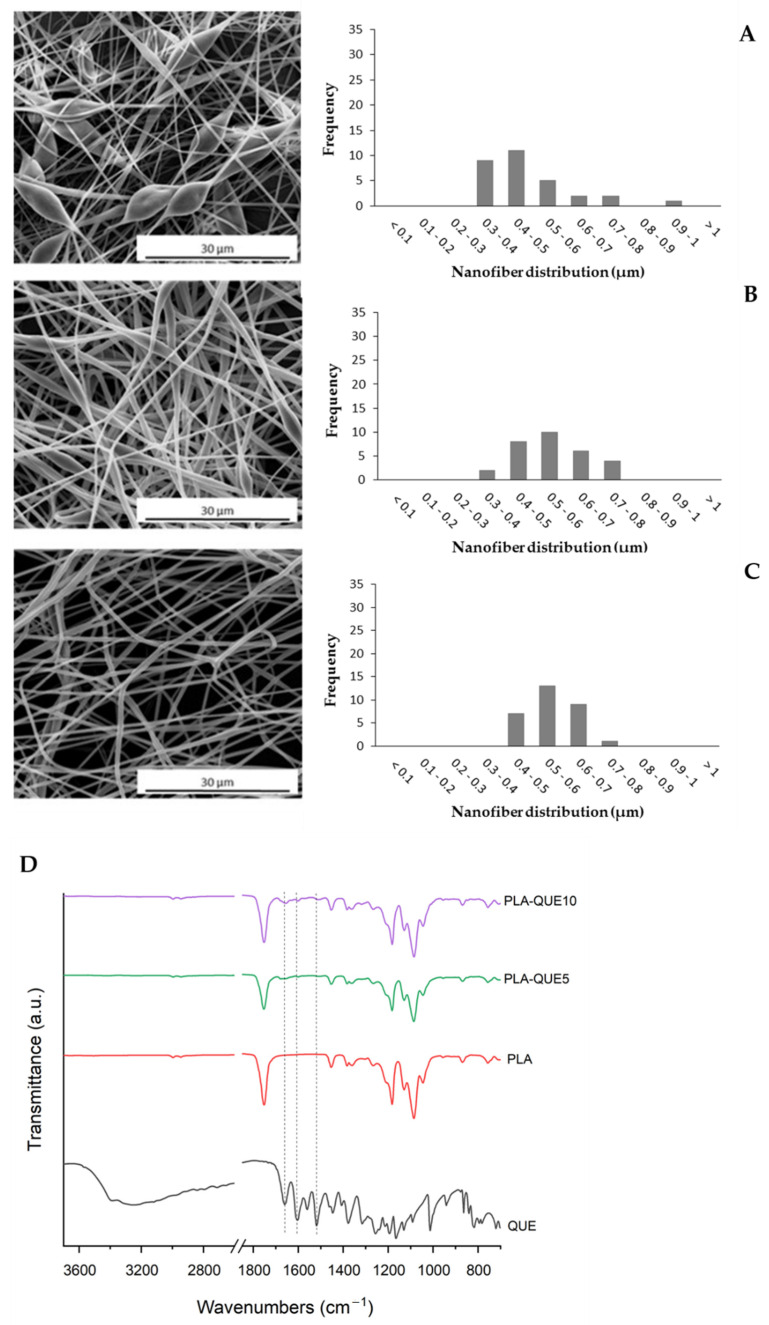
SEM micrographs (**left**) and size-distribution (**right**) of fibers prepared from polylactic acid (PLA) solutions containing various amounts of quercetin (QUE): (**A**) PLA, (**B**) PLA-QUE_5_ and (**C**) PLA-QUE_10_. (**D**) FTIR-ATR spectra of QUE, neat PLA, PLA-QUE_5_ and PLA-QUE_10_ fibers.

As shown in Figure 1, the SEM micrographs and the distribution of the diameters reveal that the PLA-QUE fibers exhibited a three-dimensional interconnected pore structure with a smooth surface (no drug particle aggregates). Some elongated beads are manifest for low QUE amounts, probably due to a slight variation in solution viscosity and surface tension, as already reported in the literature [32,33]. A monomodal diameter distribution was observed for all samples; the increasing amount of loaded QUE reduced diameter distribution and increased the average value.

The encapsulation of quercetin within the nanofiber was confirmed by ATR spectroscopy (Figure 1D). The ATR spectrum of QUE depicted typical absorption bands [34]. The most interesting peaks are those corresponding to C=O groups (1658 cm^−1^), C–C groups (1603 cm^−1^) and C=C groups (1518 cm^−1^), which are evident also in the PLA-QUE fibers (1651 cm^−1^, 1599 cm^−1^ and 1513 cm^−1^, respectively). The small shifts recorded suggest interactions between the drug and the matrix [23].

Entrapment efficiency describes the efficiency of the loading technique in incorporating drugs into the carrier system. The QUE encapsulation efficiency in the PLA fibers was 81% for PLA-QUE_5_ and 72% for PLA-QUE_10_. The decrease in encapsulation efficiency at high drug concentrations was probably due to the loss of a small part of the drug that self-aggregated, limiting its encapsulation into the fibers. Indeed, the solubility of the drug into a polymer solution is critical for the achievement of a local delivery. Thus, considering the technique employed in the fabrication of nanofiber, the drug-loading capacity was found to be efficient.

### 3.2. In Vitro Drug Release

**Figure 2:**
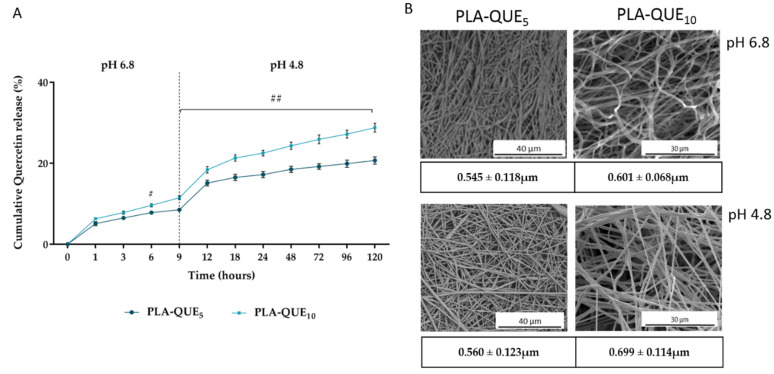
(**A**) Cumulative release profiles of quercetin at 37 °C from PLA-QUE_5_ and PLA-QUE_10_ incubated in SAGF-suc at pH 6.8 from 0 to 9 h, and at pH 4.8 from 10 to 120 h. For each sample, six different experiments were conducted, and the results are expressed as the mean of the values obtained (mean ± SD). Statistically significant variations: # *p* < 0.05, and ## *p* < 0.01 versus PLA-QUE_5_. (**B**) SEM micrographs of PLA-QUE_5_ and PLA-QUE_10_ fibers and average diameters of PLA-QUE_5_ and PLA-QUE_10_ fibers after immersion at pH 6.8 and pH 4.8.

Intra-pocket drug delivery systems have become a promising way to treat periodontitis, since they present numerous clinical, pharmacologic and toxicologic advantages over conventional treatments [35]. A periodontal pocket can act as a natural reservoir, providing a high enough concentration of active drugs for a long period of time. However, during infection, the environment tends to become acidic due to the combined action of bacterial metabolism and the host’s immune response [36,37]. To mimic the periodontal disease microenvironment characterized by subtle variations of pH level or degree of inflammation, the QUE release kinetics from PLA-membranes were determined in SAGF-suc, first at pH 6.8 (9 h) and then at pH 4.8 (111 h) simulating healthy and disease conditions, respectively (Figure 2A). 

In the absence of a stimulus, QUE release showed a burst release within the first hour, which could be attributed to the dissociation of the drug adhering to the surface of the fibers [38]. The initial burst was followed by a slow and continuous release of QUE until 9 h of incubation, reaching concentrations of ~5 µM (PLA-QUE_5_) and ~7 µM (PLA-QUE_10_), respectively. The slow quercetin release in the neutral phase is probably due to a combination of drug diffusion through the polymer matrix driven by the difference of chemical potential (concentration gradient) and a slow polymer degradation [39]. The acidification of the medium to a range within that reported for periodontal disease (pH 4.8) triggered a sudden increase in the QUE release until it reached, at the end of the experimental period, a concentration of ~13 µM for PLA-QUE_5_ and ~16 µM for PLA-QUE_10_. As previously demonstrated, PLA nanofibers act as a pH-dependent system with a remarkably high QUE release when pH decreases [24]. It should be noted that the slope of the release curve at pH 4.8 was about two times higher than that at pH 6.8, as the acidic pH induced a faster release of the drug captured in the bulk polymer fiber. Indeed, the acidic environment catalyzes the degradation of bulk material, resulting in accelerated hydrolysis of the ester linkages in the interior of the fiber with increases in drug release [40]. 

The morphological analysis carried out on the samples after the in vitro drug release experiment (Figure 2B) revealed that the fibers kept their original morphology and that the surface was still homogenous without any evidence of deterioration. Moreover, the immersion in acidic buffer resulted in a diameter increase by about 3% for PLA-QUE_5_ and 15% for PLA-QUE_10_. 

The data reported allow us to hypothesize that the higher QUE release under acidic conditions is ascribed to both the activity of the water molecules that provide the driving force for the QUE diffusion as the membrane swells under acidic conditions, and the higher QUE solubility at acidic pH. The prepared membranes were able to release QUE in a sustained manner for a time period up to 90 days in both acidic and neutral conditions until almost 80% of the encapsuled drug had been released (data not shown), demonstrating their potential use as coadjutants in periodontitis treatment.

### 3.3. Antimicrobial and Antibiofilm Activity

The antimicrobial activity of QUE-loaded membranes was assessed in growth medium supplemented with 20% sucrose by monitoring the growth rate of PAO1 and S. mutans bacteria in the presence of the prepared membranes (Table 3). The pH of the culture medium was also measured at time-point intervals, highlighting that medium acidification occurs already after the first 9 h of growth thanks to bacterial acid lactic production that leads to an environmental pH decrease [41].

The results indicate that QUE released from PLA membranes was able to inhibit only the growth of S. mutans, while no significant effect was detected for PAO1. This effect is in line with the results of J. Ouyang et al. [42], who reported a minimum inhibitory concentration (MIC) of QUE against PAO1 > 256 µg mL^−1^, a concentration that PLA membranes did not reach throughout the experimental period. 

The main pathogenesis of gingivitis and periodontitis is subgingival plaque formation caused primarily by bacteria that colonize the gingival crevice and attach to intraperiodontal pockets. Because the site of bacterial infection is usually inaccessible to agents present in the oral cavity, a local antibiofilm agent loaded in an intra-pocket delivery system promotes a high drug concentration in the gingival crevice fluid, contributing to the eradication of microbial plaque. 

To assess the PLA-QUE’s antibiofilm activity, two different biofilm analysis models were used—a static (or closed) model in which the nutrients and oxygen are provided only at the beginning of the experiment, and a dynamic (or open) model that maintains a continuous nutrient flux throughout the experimental time [43]. 

**Figure 3:**
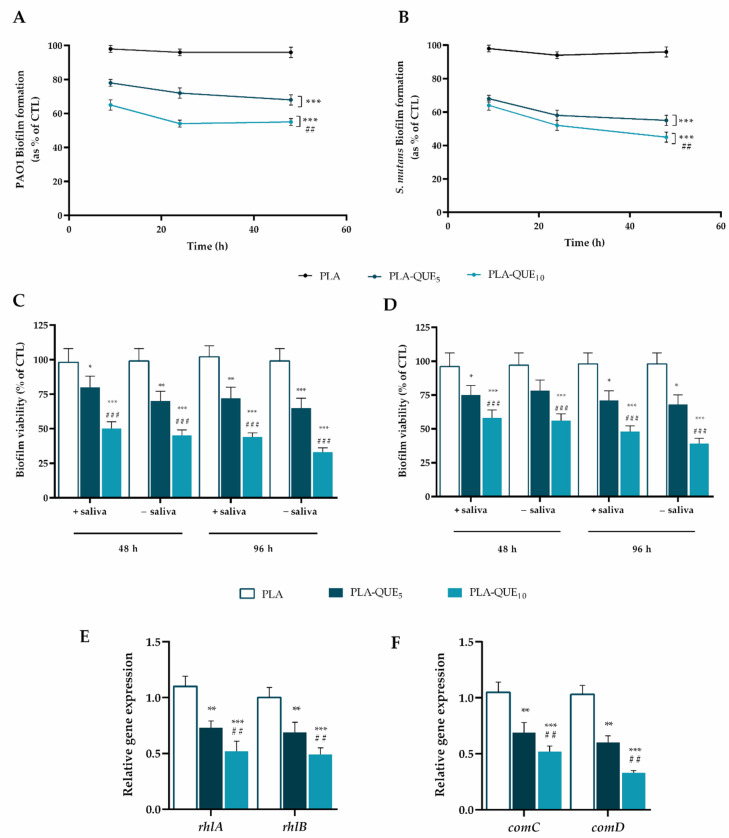
Antibiofilm activity of quercetin-loading membranes. Biofilm formation in a static mode was evaluated by the CV assay, after 6, 12 and 24 h of incubation at 37 °C in the presence of PAO1 (**A**) and *S. mutans* (**B**) measuring the absorbance at 570 nm (OD570) in a microplate reader. Bacterial growth in the presence of PLA was used as control. Biofilm formation in a dynamic mode was evaluated by the MTT assay, simulating or not simulating the formation of salivary pellicle, after 48 and 96 h of incubation at 37 °C in the presence of PAO1 (**C**) and *S. mutans* (**D**). The absorbance of each well was read at 510 nm. Biofilm formation was reported as a percentage in comparison with the maximum amount of biofilm produced by PAO1 and *S. mutans* grown in the presence of PLA (positive controls). Relative RNA expression of *rhlA* and *rhlB* in PAO1 (**E**); relative RNA expression of *comC* and *comD* in *S. mutans* (**F**). Different gene expression levels were normalized to the level of *16sRNA* gene transcripts. Gene expression levels of biofilm formed in the presence of PLA were used as control. For each sample, six different experiments were conducted, and the results are expressed as the mean of the values obtained (mean ± SD). Statistically significant variations: * *p* < 0.05, ** *p* < 0.01 and *** *p* < 0.001 versus PLA; ## *p* < 0.01 and ### *p* < 0.001 versus PLA-QUE_5_.

Crystal violet staining was used in the static biofilm model to determine the antibiofilm properties of PLA membranes after 9, 24 and 48 h of incubation in the presence of PAO1 and *S. mutans* cultures. As shown in Figure 3A,B, a significant (*p* < 0.001) antibiofilm activity was observed in both PAO1 and *S. mutans* already after 9 h of incubation, indicating a good efficacy of the released QUE in strongly inhibiting biofilm formation. Notably, PLA-QUE_10_ exhibited a significantly (*p* < 0.01) greater effect than PLA-QUE_5_ against both PAO1 and *S. mutans* strains.

A dip-flow reactor was cast off as a dynamic experimental model to recreate the oral cavity conditions where the adherence of bacteria and the consequent biofilm production are subjected to a continuous nutrient flux generating low and medium shear stress [44,45]. Moreover, the incorporation of saliva in the presence of salivary pellicle during the experiment allows for a more consistent translation of the biofilm dynamic model into an oral microcosm model. The metabolic activity of biofilms, quantified by the MTT assay, demonstrated a significant reduction in biofilm viability, regardless of the bacterial strain used and the presence or absence of salivary pellicle (Figure 3C,D). In particular, the greatest effect was observed on PAO1 at 96 h for PLA-QUE_10_ in the absence of salivary pellicle, inducing a 66% biofilm inhibition in comparison with the PLA (*p* < 0.001). Furthermore, the presence of salivary pellicle did not significantly affect the antibiofilm properties of PLA-QUE membranes for every bacterial strain.

Quorum sensing (QS) is the key bacterial mechanism of biofilm development. It is regulated by different signaling molecules, named autoinducers, that are secreted in the extracellular matrix according to bacterial density both in Gram-negative and Gram-positive bacteria [46]. Therefore, to investigate the mechanism of action of the QUE released from membranes in the inhibition of biofilm formation/maturation, the expression level of several virulence genes was evaluated by q-PCR. 

In particular, the rhlAB operon, involved in rhamnolipids synthesis, and the ComAB/ComCDE operon, codifying the signal transduction system related to the production of virulence factors, were used as target QS genes in PAO1 and *S. mutans*, respectively [19].

As reported in Figure 3E,F, PLA-QUE membranes significantly (*p* < 0.0 1, and *p* < 0.001) decreased the mRNA levels of all genes tested, as compared with the PLA membrane. In particular, the incubation of PAO1 in the presence of PLA-QUE_10_ decreased the relative expression levels of the rhlA and rhlB genes 0.52- and 0.49-fold, respectively. Similarly, a 0.54- and 0.33-fold reduction in the relative expression levels of *comC* and *comD*, respectively, was observed for *S. mutans* incubated in the presence of PLA-QUE_10_. These results indicate that QUE may inhibit biofilm formation by triggering the expression levels of key genes involved in QS.

### 3.4. QUE-Loaded Membranes Inhibit LPS-Induced Antioxidant Activity

One of the goals in periodontal treatment is to control or eliminate inflammatory reactions and counteract local and systemic oxidative stress. Indeed, the persistence of bacterial pathogens on the dental surface and in the surrounding periodontal pockets triggers an aberrant defense response by the host that leads, if not properly treated, to periodontal tissue destruction. In periodontally healthy conditions, ROS contribute to the oxidative killing of pathogens, while, during periodontitis, the homeostatic imbalance between ROS and antioxidant defense systems acts as intracellular signal transducers that promote cell death and cause tissue damage. Several studies have demonstrated the capability of QUE to reduce either in vitro and in vivo oxidative stress and inflammation [47,48,49,50,51]. In particular, the antioxidant effects of QUE seem to be related to the presence in a heterocyclic ring of a catechol group (a 2,3-double bond at position 3 and a hydroxyl substitution at position 5) [50,52]. Gómez-Florit and colleagues identified QUE among all the screened flavonoids as the most promising molecule for periodontal disease treatment [53]. Their results demonstrated that QUE decreased ROS levels in gingival fibroblasts under both basal and stimulated conditions, protecting the integrity of gingival tissues. Wei et al. provided evidence of the ability of QUE to alleviate oxidative damage and enhance the antioxidant capacity of periodontal ligament cells through the activation of the NF-E2–related factor 2 (NRF2) signaling pathway. In addition, quercetin reduced the oxidative stress levels of mice with periodontitis [14]. 

**Figure 4:**
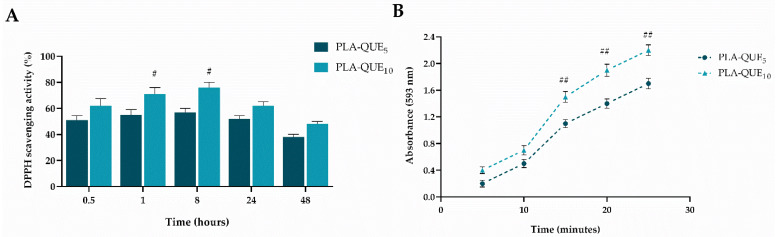
DPPH scavenging activity (**A**) and ferric reducing power (FRAP assay) (**B**) of QUE-loading membranes at different QUE concentrations (5% and 10%). For each sample, six different experiments were carried out, and the results are expressed as the mean of the values obtained (mean ± SD). Statistically significant variations: # *p* ≤ 0.05 and ## *p* < 0.01 versus PLA-QUE_5_.

To establish the antioxidant ability of QUE released from PLA-membranes against various reactive oxygen species, such as DPPH radical or metal ions, cell-free systems were used. The DPPH assay measures the ability of a bioactive molecule to inhibit lipid oxidation, while the FRAP assay is based on electron-transfer reactions in which a molecule is able to reduce Fe^3+^-tripyridyltriazine to the colored Fe^2+^-tripyridyltriazine. As reported in Figure 4A, a decrease in the DPPH radical concentration was shown due to the scavenging ability of quercetin released from PLA-QUE at different concentrations (5%, and 10%). However, the PLA-QUE_10_ showed a significantly greater effect (*p* < 0.05) than PLA-QUE_5_. Moreover, the reduction in antioxidant activity registered after the longer immersion period (48 h) could be probably related to the decreasing stability of quercetin in alcohol solution. The ability of PLA-QUE_5_ to reduce iron ions was significantly lower (*p* < 0.01) when compared to that of PLA-QUE_10_ (Figure 4B). The antioxidant results confirm the ability of released QUE to react with free radicals or terminate chain reactions, corroborating the capacity of electrospinning technology to provide greater protection of bioactive compounds.

**Figure 5:**
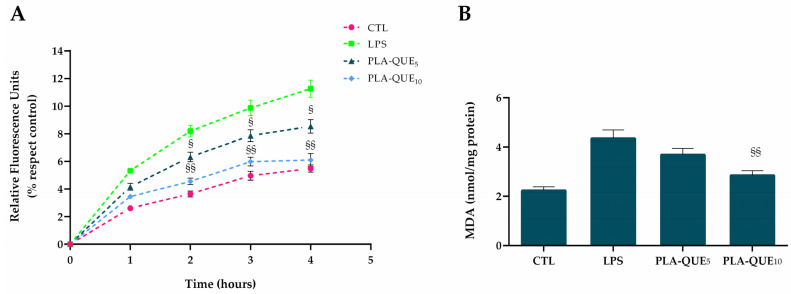
QUE reduced the production of reactive oxygen species (ROS) and lipid peroxidation in LPS-stimulated human gingival fibroblasts (HGF). (**A**) ROS production was determined by the DCF fluorescence intensity using a microplate reader. (**B**) Malondialdehyde was used as a marker of lipid peroxidation. Treatment with LPS of *Porphyromonas gingivalis* (1 μg/mL) is a positive control. Each graph represents three independent experiments, and the data are expressed as mean ± S.D. Statistically significant variations: § *p* < 0.05 and §§ *p* < 0.01 versus LPS.

The efficiency of QUE-loaded nanofibers in reducing intracellular ROS generation in HGFs was verified using DCFH-DA and MDA assay kits (Figure 5). The DCFH-DA assay relies on the ability of redox-active molecules to inhibit or promote the oxidation of the probe absorbed by cells into its fluorescent form [54]. After incubation with LPS, PLA-QUE membranes significantly decreased LPS-induced formation of oxidants (Figure 5A). In particular, the pre-treatment of cells with PLA-QUE_10_ induced an evident decrease in ROS production of about 47%, while in the case of PLA-QUE_5_ the inhibition settled around 27%. As reported in Figure 5B, a marked increase in the MDA level was observed in cells treated with LPS compared to the untreated group, whereas pre-treatment with QUE-loading membranes produced a statistically significant decrease in lipoperoxidation. Indeed, excessive lipoperoxidation damages cell membranes and intracellular organelles, impairing cell metabolism. Hence, the accumulation of toxic metabolites not only affects periodontal tissues, but also leads to significant metabolic disorders in the entire body [55]. 

### 3.5. Anti-Inflammatory Effects of PLA-QUE on LPS-Stimulated HGFs

**Figure 6:**
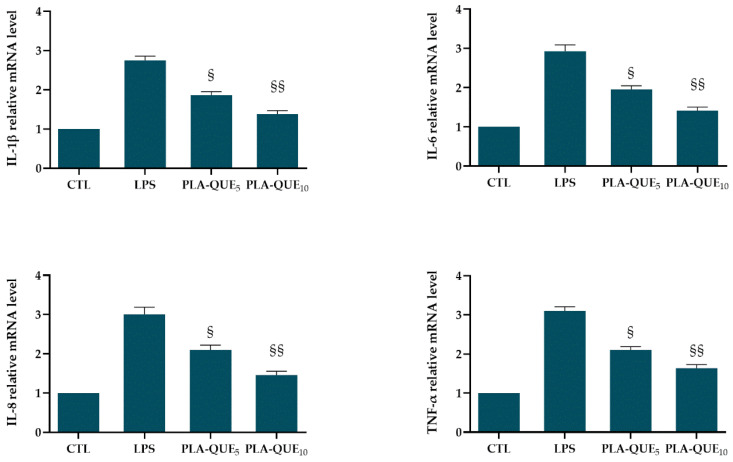
Effects of released QUE on mRNA levels of *IL-1β*, *IL-6*, *IL-8* and *TNF-α* in LPS-stimulated HGFs. HGFs were pre-treated with PLA-QUE membranes for 24 h, followed by LPS stimulation (final concentration at 1 μg/mL) for 3 h. The values of three independent experiments are presented as means ± SD. § *p* < 0.05 and §§ *p* < 0.01 versus LPS.

Current non-surgical therapy for periodontitis intends to remove the bacterial biofilm and the tartar above and below the gingival area, limiting the charged bacterial periodontal pathogens. However, the presence of bacteria was only regarded as the initiating factor. Indeed, the pathogenesis of periodontitis involves several molecules of the immune system that interact in a network to eliminate the pathogens, themselves contributing to noticeable damage in the whole apparatus of the periodontium [56,57]. Within the progression of periodontitis, there is high expression of proinflammatory cytokines such as interleukin IL-1β, IL-6, IL-8, tumor necrosis factor (TNF)-α and regulatory cytokines such as IL-4. Cytokines are hormone-like proteins produced by resident cells (epithelial and fibroblasts) and phagocytes in the early chronic phases of inflammation. These peptides act in the first wave of responses against pathogens and stimuli at barrier sites and connect tissue cells with lymphocytes and accessory cell populations [58]. Several studies report the upregulation of interleukins in the gingival crevicular fluid, saliva and gingival tissues of periodontal patients compared with healthy individuals [59]. In particular, the inflammatory mediators in whole saliva are derived not only from the periodontium via the influx of gingival crevicular fluid but also from the mucosa during the progression of periodontal disease [11]. Indeed, HGFs, the major constituents of gingival connective tissue, recognize LPS and mediate host immune response by initiating an inflammatory response, including the production of various proinflammatory cytokines such as interleukin IL-1, IL-6 and TNF-α [60,61]. IL-1β participates in inflammation, immune regulation and bone resorption, inducing the production of IL-6. Its overproduction may initiate and facilitate the breakdown of connective tissue driven by proteinases [62]. As a major chemoattractant, IL-8 can recruit neutrophils which can cause the destruction of normal periodontal tissues by releasing metalloproteinases. TNF-α, generated in the early period of inflammation, causes oxidative damage to periodontal tissues due to its effectiveness in inducing superoxide production in HGFs. Therefore, bioactive molecules able to suppress these inflammatory mediators or block the signaling pathway involved may help to reduce the pathological process [63,64]. In the present study, the mRNA levels of IL-1β, IL-6, IL-8 and TNF-α were assessed on LPS-stimulated HGFs by RT-qPCR (Figure 6). The release of the aforementioned inflammatory mediators was induced by stimulating HGFs with *P. gingivalis* LPS. Indeed, the Gram-negative bacterium *P. gingivalis* represents a crucial virulence factor for periodontitis [65,66]. As expected, the levels of all tested cytokines (relative to the housekeeping gene) were significantly upregulated in LPS-treated cells, compared with the control group. These inflammatory mediators can be downregulated effectively by both PLA-QUE membranes, corroborating the anti-inflammatory capability of released QUE. In particular, PLA-QUE_10_ led to an approximately 50% decrease in interleukin (IL-1β, IL-6 and IL-8) and TNF-α expression levels compared to LPS-treated cells. The reduction in the inflammatory process was higher using PLA-QUE_10_ than PLA-QUE_5_ due to a concentration-dependent issue.

### 3.6. Conclusions

Because periodontal disease is an inflammatory disease closely related to bacterial infection, its management requires the synergistic use of both antibiofilm and anti-inflammatory drugs to improve the curative effect. In the present study, electrospun PLA nanofibers loaded with different amounts of QUE were produced and characterized and their antibacterial and antibiofilm potential evaluated on PAO1 and *S. mutans*, which are responsible for both acute and chronic infections in humans. The reported results demonstrated that PLA-QUE membranes can act as a drug reservoir at neutral pH, releasing high QUE concentrations only at pH < 5.5 (such as in the presence of oral bacterial infection), and thus limiting PAO1 and *S. mutans* biofilm maturation. It should be noted that the slope of the release curve at pH 4.8 was about two times higher than that at pH 6.8, as the acidic pH induced the faster release of the drug captured in the bulk PLA fibers.

Furthermore, the pre-treatment of *P. gingivalis* LPS-stimulated HGFs with PLA-QUE_10_ induced an evident decrease in ROS production of about 47%, while in the case of PLA-QUE_5_ the inhibition settled around 27% compared to the untreated group. Moreover, a marked increase in the MDA level was observed in cells treated with LPS, whereas pre-treatment with QUE-loading membranes produced a statistically significant decrease in lipoperoxidation.

Finally, PLA-QUE membranes elicited in vitro anti-inflammatory activity and were able to effectively downregulate selected inflammatory mediators. In particular, PLA-QUE_10_ led to an approximate 50% decrease in interleukin (IL-1β, IL-6 and IL-8) and TNF-α expression levels compared to LPS-treated cells. The reduction in the inflammatory process was higher using PLA-QUE_10_ than PLA-QUE_5_ due to a concentration-dependent issue.

The reported results demonstrated that PLA-QUE membranes can represent an interesting solution as adjuvants in the local treatment of periodontal disease. 

## Figures and Tables

**Table 1:** qRT-PCR primers.

Gene	Forward Primer (5′–3′)	Reverse Primer (5′–3′)
*rhlA*	AGCTGGGACGAATACACCA	GACTCCAGGTCGAGGAAATG
*rhlB*	GAGCGACGAACTGACCTACC	GTTGAACTTGGGGTGTACCG
*comC*	GACTTTAAAGAAATTAAGACTG	AAGCTTGTGTAAAACTTCTGT
*comD*	CTCTGATTGACCATTCTTCTGG	CATTCTGAGTTTATGCCCCTC
*16SrRNA*	CCTACGGGAGGCAGCAGTAG	CAACAGAGCTTTACGATCCGAAA
*16SrRNA*	CAAAACTACTGAGCTAGAGTACG	TAAGATCTCAAGGATCCCAACGGCT

**Table 2:** Primers used for qRT-PCR.

Gene	Forward Primer (5′–3′)	Reverse Primer (5′–3′)
*IL-1β*	TAGGGCTGGCAGAAAGGGAACA	GTGGGAGCGAATGACAGAGGGT
*IL-6*	CGCCTTCGGTCCAGTTGCC	GCCAGTGCCTCTTTGCTGCTTT
*IL-8*	CTCTTGGCAGCCTTCCTGATTTC	TTTTCCTTGGGGTCCAGACAGAG
*TNF-α*	AACATCCAACCTTCCCAAACGC	TGGTCTCCAGATTCCAGATGTCAGG
*β-actin*	GACTTAGTTGCGTTACACCCTTTCTTG	CTGTCACCTTCACCGTTCCAGTTTT

**Table 3:** pH and OD 600 nm of the bacterial cultures in growth medium supplemented with 20% sucrose in the presence of PLA membranes.

***Pseudomonas aeruginosa***						
**Time**	**PLA**		**PLA-QUE_5_**		**PLA-QUE_10_**	
	pH	OD600 nm	pH	OD600 nm	pH	OD600 nm
9 h	5.82 ± 0.33	1.35 ± 0.06	5.85 ± 0.28	1.37 ± 0.7	5.80 ± 0.38	1.33 ± 0.06
24 h	5.02 ± 0.29	1.60 ± 0.07	4.95 ± 0.32	1.55 ± 0.06	5.03 ± 0.35	1.61 ± 0.07
48 h	4.90 ± 0.46	1.65 ± 0.08	4.88 ± 0.43	1.61 ± 0.06	4.91 ± 0.37	1.66 ± 0.09
96 h	4.83 ± 0.38	1.71 ± 0.06	4.86 ± 0.35	1.66 ± 0.07	4.81 ± 0.29	1.69 ± 0.07
***Streptococcus mutans***						
**Time**	**PLA**		**PLA-QUE_5_**		**PLA-QUE_10_**	
	pH	OD600 nm	pH	OD600 nm	pH	OD600 nm
9 h	5.66 ± 0.45	0.55 ± 0.06	5.73 ± 0.35	0.35 ± 0.03	5.75 ± 0.41	0.31 ± 0.03
24 h	4.93 ± 0.39	0.99 ± 0.09	4.98 ± 0.43	0.77 ± 0.03	4.99 ± 0.45	0.56 ± 0.04
48 h	4.85 ± 0.36	1.35 ± 0.06	4.84 ± 0.29	1.24 ± 0.07	4.84 ± 0.34	1.03 ± 0.06
96 h	4.79 ± 0.45	1.53 ± 0.07	4.81 ± 0.37	1.33 ± 0.06	4.82 ± 0.39	1.12 ± 0.07

## Data Availability

Not applicable.

## Sample Availability

Samples are available on reasonable request from the corresponding authors.

## References

[1] Tonetti M.S., Eickholz P., Loos B.G., Papapanou P., van der Velden U., Armitage G., Bouchard P., Deinzer R., Dietrich T., Hughes F. (2015). Principles in prevention of periodontal diseases: Consensus report of group 1 of the 11th European Workshop on Periodontology on effective prevention of periodontal and peri-implant diseases. J. Clin. Periodontol..

[2] Kononen E., Gursoy M., Gursoy U.K. (2019). Periodontitis: A Multifaceted Disease of Tooth-Supporting Tissues. J. Clin. Med..

[3] Kinane D.F., Stathopoulou P.G., Papapanou P.N. (2017). Periodontal diseases. Nat. Rev. Dis. Primers.

[4] Demmer R.T., Papapanou P.N. (2010). Epidemiologic patterns of chronic and aggressive periodontitis. Periodontol. 2000.

[5] Genco R.J., Sanz M. (2020). Clinical and public health implications of periodontal and systemic diseases: An overview. Periodontol. 2000.

[6] Schenkein H.A., Papapanou P.N., Genco R., Sanz M. (2020). Mechanisms underlying the association between periodontitis and atherosclerotic disease. Periodontol. 2000.

[7] Petersen P.E., Ogawa H. (2005). Strengthening the prevention of periodontal disease: The WHO approach. J. Periodontol..

[8] Tonetti M.S., D’Aiuto F., Nibali L., Donald A., Storry C., Parkar M., Suvan J., Hingorani A.D., Vallance P., Deanfield J. (2007). Treatment of periodontitis and endothelial function. N. Engl. J. Med..

[9] Graziani F., Karapetsa D., Alonso B., Herrera D. (2017). Nonsurgical and surgical treatment of periodontitis: How many options for one disease?. Periodontol. 2000.

[10] Taheri J.B., Azimi S., Rafieian N., Zanjani H.A. (2011). Herbs in dentistry. Int. Dent. J..

[11] Ara T., Nakatani S., Kobata K., Sogawa N., Sogawa C. (2018). The Biological Efficacy of Natural Products against Acute and Chronic Inflammatory Diseases in the Oral Region. Medicines.

[12] Yang D., Wang T., Long M., Li P. (2020). Quercetin: Its Main Pharmacological Activity and Potential Application in Clinical Medicine. Oxid. Med. Cell. Longev..

[13] Elnagdy S., Raptopoulos M., Kormas I., Pedercini A., Wolff L.F. (2021). Local Oral Delivery Agents with Anti-Biofilm Properties for the Treatment of Periodontitis and Peri-Implantitis. A Narrative Review. Molecules.

[14] Wei Y., Fu J., Wu W., Ma P., Ren L., Yi Z., Wu J. (2021). Quercetin Prevents Oxidative Stress-Induced Injury of Periodontal Ligament Cells and Alveolar Bone Loss in Periodontitis. Drug Des. Devel..

[15] Zhang W., Jia L., Zhao B., Xiong Y., Wang Y.N., Liang J., Xu X. (2021). Quercetin reverses TNFalpha induced osteogenic damage to human periodontal ligament stem cells by suppressing the NFkappaB/NLRP3 inflammasome pathway. Int. J. Mol. Med..

[16] Taskan M.M., Gevrek F. (2020). Quercetin Decreased Alveolar Bone Loss and Apoptosis in Experimentally Induced Periodontitis Model in Wistar Rats. Antiinflamm. Antiallergy Agents Med. Chem..

[17] He Z., Zhang X., Song Z., Li L., Chang H., Li S., Zhou W. (2020). Quercetin inhibits virulence properties of Porphyromas gingivalis in periodontal disease. Sci. Rep..

[18] Mooney E.C., Holden S.E., Xia X.J., Li Y., Jiang M., Banson C.N., Zhu B., Sahingur S.E. (2021). Quercetin Preserves Oral Cavity Health by Mitigating Inflammation and Microbial Dysbiosis. Front. Immunol..

[19] Di Salle A., Viscusi G., Di Cristo F., Valentino A., Gorrasi G., Lamberti E., Vittoria V., Calarco A., Peluso G. (2021). Antimicrobial and Antibiofilm Activity of Curcumin-Loaded Electrospun Nanofibers for the Prevention of the Biofilm-Associated Infections. Molecules.

[20] Zhuang Y., Lin K., Yu H. (2019). Advance of Nano-Composite Electrospun Fibers in Periodontal Regeneration. Front. Chem..

[21] Budai-Szucs M., Ruggeri M., Faccendini A., Leber A., Rossi S., Varga G., Bonferoni M.C., Valyi P., Burian K., Csanyi E. (2021). Electrospun Scaffolds in Periodontal Wound Healing. Polymers.

[22] Wang Y., Liu Y., Zhang X., Liu N., Yu X., Gao M., Wang W., Wu T. (2021). Engineering Electrospun Nanofibers for the Treatment of Oral Diseases. Front. Chem..

[23] Baliga S., Muglikar S., Kale R. (2013). Salivary pH: A diagnostic biomarker. J. Indian Soc. Periodontol..

[24] Bonadies I., Di Cristo F., Valentino A., Peluso G., Calarco A., Di Salle A. (2020). pH-Responsive Resveratrol-Loaded Electrospun Membranes for the Prevention of Implant-Associated Infections. Nanomaterials.

[25] Stoyanova N., Spasova M., Manolova N., Rashkov I., Georgieva A., Toshkova R. (2020). Antioxidant and Antitumor Activities of Novel Quercetin-Loaded Electrospun Cellulose Acetate/Polyethylene Glycol Fibrous Materials. Antioxidants.

[26] Amrati F.E., Bourhia M., Slighoua M., Ibnemoussa S., Bari A., Ullah R., Amaghnouje A., Di Cristo F., El Mzibri M., Calarco A. (2020). Phytochemical Study on Antioxidant and Antiproliferative Activities of Moroccan Caralluma europaea Extract and Its Bioactive Compound Classes. Evid. -Based Complement. Altern. Med..

[27] Mayer S., Tallawi M., De Luca I., Calarco A., Reinhardt N., Gray L.A., Drechsler K., Moeini A., Germann N. (2021). Antimicrobial and physicochemical characterization of 2,3-dialdehyde cellulose-based wound dressings systems. Carbohydr. Polym..

[28] Tammaro L., Salle A.D., Calarco A., Luca I., Riccitiello F., Peluso G., Vittoria V., Sorrentino A. (2020). Multifunctional Bioactive Resin for Dental Restorative Materials. Polymers.

[29] Kost B., Svyntkivska M., Brzeziński M., Makowski T., Piorkowska E., Piorkowska E., Rajkowska K., Kunicka-Styczyńska A., Biela T. (2020). PLA/β-CD-based fibres loaded with quercetin as potential antibacterial dressing materials. Colloids Surf. B Biointerfaces.

[30] Ajmal G., Bonde G.V. (2019). Ciprofloxacin HCl and quercetin functionalized electrospun nanofiber membrane: Fabrication and its evaluation in full thickness wound healing. Artif. Cells Nanomed. Biotechnol..

[31] Vashisth P., Singh R.P., Pruthi V. (2015). A controlled release system for quercetin from biodegradable poly(lactide-co-glycolide)–polycaprolactone nanofibers and its in vitro antitumor activity. J. Bioact. Compat. Polym..

[32] Bonadies I., Ambrogi V., Ascione L., Carfagna C. (2014). A hyperbranched polyester as antinucleating agent for Artemisinin in electrospun nanofibers. Eur. Polym. J..

[33] Peng J., Qian Z., Wang B., Fu S., Guo G., Luo F., Li R., Wu D. (2011). Preparation and release characteristic of quercetin loaded poly(lactic acid) ultrafine fibers. J. Nanosci. Nanotechnol..

[34] Vashisth P., Nikhil K., Pemmaraju S.C., Pruthi P.A., Mallick V., Singh H., Patel A., Mishra N.C., Singh R.P., Pruthi V. (2013). Antibiofilm activity of quercetin-encapsulated cytocompatible nanofibers against Candida albicans. J. Bioact. Compat. Polym..

[35] Wei Y., Deng Y., Ma S., Ran M., Jia Y., Meng J., Han F., Gou J., Yin T., He H. (2021). Local drug delivery systems as therapeutic strategies against periodontitis: A systematic review. J. Control. Release.

[36] Lamont R.J., Koo H., Hajishengallis G. (2018). The oral microbiota: Dynamic communities and host interactions. Nat. Rev. Microbiol..

[37] Olsen I., Lambris J.D., Hajishengallis G. (2017). Porphyromonas gingivalis disturbs host-commensal homeostasis by changing complement function. J. Oral Microbiol..

[38] Toncheva A., Paneva D., Manolova N., Rashkov I. (2011). Electrospun poly(L-lactide) membranes containing a single drug or multiple drug system for antimicrobial wound dressings. Macromol. Res..

[39] Li Z., Mei S., Dong Y., She F., Li Y., Li P., Kong L. (2020). Functional Nanofibrous Biomaterials of Tailored Structures for Drug Delivery-A Critical Review. Pharmaceutics.

[40] Wang Y., Murcia Valderrama M.A., van Putten R.J., Davey C.J.E., Tietema A., Parsons J.R., Wang B., Gruter G.M. (2021). Biodegradation and Non-Enzymatic Hydrolysis of Poly(Lactic-co-Glycolic Acid) (PLGA12/88 and PLGA6/94). Polymers.

[41] Cavazana T.P., Pessan J.P., Hosida T.Y., Monteiro D.R., Botazzo Delbem A.C. (2018). pH changes of mixed biofilms of Streptococcus mutans and Candida albicans after exposure to sucrose solutions in vitro. Arch. Oral Biol..

[42] Ouyang J., Sun F., Feng W., Sun Y., Qiu X., Xiong L., Liu Y., Chen Y. (2016). Quercetin is an effective inhibitor of quorum sensing, biofilm formation and virulence factors in Pseudomonas aeruginosa. J. Appl. Microbiol..

[43] Guzman-Soto I., McTiernan C., Gonzalez-Gomez M., Ross A., Gupta K., Suuronen E.J., Mah T.F., Griffith M., Alarcon E.I. (2021). Mimicking biofilm formation and development: Recent progress in in vitro and in vivo biofilm models. iScience.

[44] Zaltsman N., Ionescu A.C., Weiss E.I., Brambilla E., Beyth S., Beyth N. (2017). Surface-modified nanoparticles as anti-biofilm filler for dental polymers. PLoS ONE.

[45] Guggenheim B., Giertsen E., Schupbach P., Shapiro S. (2001). Validation of an in vitro biofilm model of supragingival plaque. J. Dent. Res..

[46] Memariani H., Memariani M., Ghasemian A. (2019). An overview on anti-biofilm properties of quercetin against bacterial pathogens. World J. Microbiol. Biotechnol..

[47] Wadia R. (2020). Periodontitis and peri-implantitis. Br. Dent. J..

[48] Boots A.W., Haenen G.R., Bast A. (2008). Health effects of quercetin: From antioxidant to nutraceutical. Eur. J. Pharm..

[49] Li Y., Yao J., Han C., Yang J., Chaudhry M.T., Wang S., Liu H., Yin Y. (2016). Quercetin, Inflammation and Immunity. Nutrients.

[50] Xu D., Hu M.J., Wang Y.Q., Cui Y.L. (2019). Antioxidant Activities of Quercetin and Its Complexes for Medicinal Application. Molecules.

[51] Ha A.T., Rahmawati L., You L., Hossain M.A., Kim J.H., Cho J.Y. (2021). Anti-Inflammatory, Antioxidant, Moisturizing, and Antimelanogenesis Effects of Quercetin 3-O-beta-D-Glucuronide in Human Keratinocytes and Melanoma Cells via Activation of NF-kappaB and AP-1 Pathways. Int. J. Mol. Sci..

[52] Kim B.H., Choi J.S., Yi E.H., Lee J.K., Won C., Ye S.K., Kim M.H. (2013). Relative antioxidant activities of quercetin and its structurally related substances and their effects on NF-kappaB/CRE/AP-1 signaling in murine macrophages. Mol. Cells.

[53] Gomez-Florit M., Monjo M., Ramis J.M. (2015). Quercitrin for periodontal regeneration: Effects on human gingival fibroblasts and mesenchymal stem cells. Sci. Rep..

[54] Wolfe K.L., Liu R.H. (2007). Cellular antioxidant activity (CAA) assay for assessing antioxidants, foods, and dietary supplements. J. Agric. Food Chem..

[55] Kilmukhametova Y.H., Batig V.M., Ostafiichuk M.A., Tokar O.M., Glushchenko T.A., Batih I.V., Sheremet M.I. (2021). Indicators of antioxidant protection of blood in necrotizing ulcerative gingivitis in experimental animals. J. Med. Life.

[56] Ramadan D.E., Hariyani N., Indrawati R., Ridwan R.D., Diyatri I. (2020). Cytokines and Chemokines in Periodontitis. Eur. J. Dent..

[57] Cobb C.M., Sottosanti J.S. (2021). A re-evaluation of scaling and root planing. J. Periodontol..

[58] Kumaresan D., Balasundaram A., Naik V.K., Appukuttan D.P. (2016). Gingival crevicular fluid periostin levels in chronic periodontitis patients following nonsurgical periodontal treatment with low-level laser therapy. Eur. J. Dent..

[59] Kinane D.F., Zhang P., Benakanakere M., Singleton J., Biesbrock A., Nonnenmacher C., He T. (2015). Experimental gingivitis, bacteremia and systemic biomarkers: A randomized clinical trial. J. Periodontal Res..

[60] Liu J., Wang Y., Ouyang X. (2014). Beyond toll-like receptors: Porphyromonas gingivalis induces IL-6, IL-8, and VCAM-1 expression through NOD-mediated NF-kappaB and ERK signaling pathways in periodontal fibroblasts. Inflammation.

[61] Li X., Wang X., Luan Q.X. (2021). Hyperresponsiveness of human gingival fibroblasts from patients with aggressive periodontitis against bacterial lipopolysaccharide. Exp. Med..

[62] Garlet G.P. (2010). Destructive and protective roles of cytokines in periodontitis: A re-appraisal from host defense and tissue destruction viewpoints. J. Dent. Res..

[63] Hou L.T., Liu C.M., Rossomando E.F. (1995). Crevicular interleukin-1 beta in moderate and severe periodontitis patients and the effect of phase I periodontal treatment. J. Clin. Periodontol..

[64] Yu H., Sun C., Argraves K.M. (2016). Periodontal inflammation and alveolar bone loss induced by Aggregatibacter actinomycetemcomitans is attenuated in sphingosine kinase 1-deficient mice. J. Periodontal Res..

[65] Lin F.Y., Hsiao F.P., Huang C.Y., Shih C.M., Tsao N.W., Tsai C.S., Yang S.F., Chang N.C., Hung S.L., Lin Y.W. (2014). Porphyromonas gingivalis GroEL induces osteoclastogenesis of periodontal ligament cells and enhances alveolar bone resorption in rats. PLoS ONE.

[66] Staff P.O. (2017). Correction: 3LPS-binding protein and its interactions with P. gingivalis LPS modulate pro-inflammatory response and Toll-like receptor signaling in human oral keratinocytes. PLoS ONE.

